# Complete Genome Sequences of Streptococcus sobrinus SL1 (ATCC 33478 = DSM 20742), NIDR 6715-7 (ATCC 27351), NIDR 6715-15 (ATCC 27352), and NCTC 10919 (ATCC 33402)

**DOI:** 10.1128/MRA.00804-18

**Published:** 2018-07-26

**Authors:** Mia J. Sales, William G. Herbert, Yuting Du, Amitha S. Sandur, Naaman M. Stanley, Paul A. Jensen

**Affiliations:** a Department of Bioengineering, University of Illinois at Urbana-Champaign, Urbana, Illinois, USA; b Carl R. Woese Institute for Genomic Biology, University of Illinois at Urbana-Champaign, Urbana, Illinois, USA; University of Delaware

## Abstract

The bacterium Streptococcus sobrinus causes tooth decay in humans. We present complete circularized genome sequences for four strains of S. sobrinus, type strain SL1, strain NIDR 6715-7 and the related NIDR 6715-15, and strain NCTC 10919. The finished genomes will enable genomic comparisons between S. sobrinus and other cariogenic microbes.

## Abstract (precis)

The bacterium Streptococcus sobrinus causes tooth decay in humans. We present complete circularized genome sequences for four strains of S. sobrinus, type strain SL1, strain NIDR 6715-7 and the related NIDR 6715-15, and strain NCTC 10919.

## ANNOUNCEMENT

Tooth decay, or dental caries, occurs when acid produced by oral bacteria demineralizes the tooth structure. The primary causative agents of caries are the Gram-positive mutans streptococci, including Streptococcus sobrinus (1). S. sobrinus occurs in 10 to 14% of carious lesions (2) and is associated with the poorest clinical outcomes, especially among children (3, 4).

No complete or reference genome exists for any S. sobrinus strain. The current draft genome for the S. sobrinus type strain SL1 contains 54 contigs (5). There is no genome sequence information for strain NIDR 6715 or NCTC 10919 despite multiple phenotypic studies on these microbes (6, 7). We believe that the complete S. sobrinus genomes presented herein are an important step toward understanding the genetic basis of cariogenesis by S. sobrinus.

All strains were cultured overnight in Todd-Hewitt broth with 5% yeast extract (Sigma) and 5 μl/ml Oxyrase (Oxyrase, Inc.) in sealed 15-ml tubes. Genomic DNA was extracted using an UltraClean microbial DNA isolation kit (Mo Bio), with 15 min of incubation at 65°C following step 4. DNA quantity and quality were checked using a Qubit fluorimeter (Invitrogen) and gel electrophoresis.

Genome sequencing used both Illumina short-read and Oxford Nanopore long-read sequences. Libraries for the Illumina MiSeq instrument were generated with Nextera kits and sequenced using a paired end 2 × 250-bp version 2 flow cell yielding 27.4 million reads. FASTQ files containing reads were generated and demultiplexed using blc2fastq version 2.17.1.14 (Illumina).

Nanopore sequencing libraries (8) were prepared for strains SL1, NIDR 6715-7, and NCTC 10919 using the rapid barcoding library kit SQK-RBK004 (Oxford Nanopore). Initial assembly of the Illumina reads revealed that strains NIDR 6717-7 and NIDR 6715-15 are nearly identical, so NIDR 6715-15 was not used for Nanopore sequencing. Libraries were sequenced on a single SpotON R9.4.1 FLO-MIN106 flow cell for 24 h using a GridIONx5 sequencer yielding 2.47 Gb across 537,986 reads (max length, 46.479 kb; mean length, 4.572 kb). Base calling was performed with Albacore 2.1.7 (Oxford Nanopore), and all reads shorter than 1 kb were discarded.

All Illumina short reads were trimmed to 150 bp and quality filtered using BBDuk version 37.09 (option “maq = 6”). Nanopore adapters were trimmed with PoreChop version 0.2.1 using the default settings. Illumina and Nanopore reads from strains SL1, 6715-7, and 10919 were assembled using Unicycler version 0.4.2 (9) with the default options. Each genome assembled to a single circularized contig. Hybrid assembly with SPAdes version 3.11.1 (10) resulted in 1, 1, and 3 contigs for strains SL1, NIDR 6715-7, and NCTC 10919, respectively. Assembly of the strain NIDR 6715-15 Illumina short reads with SPAdes version 3.11.1 (option “–careful”) generated 53 contigs. The contigs from strain NIDR 6715-15 were mapped to the complete NIDR 6715-7 assembly using Quast version 4.6.1 (11), revealing >99% identity between the two substrains. The NIDR 6715-15 contigs were scaffolded into a complete circularized assembly using the NIDR 6715-7 genome.

Genomes were annotated using the NCBI Prokaryotic Annotation Pipeline (12). The SL1 genome is 2.199 Mb (43.5% G+C content) with 2,094 predicted genes. The genomes for strains NIDR 6715-7 and NIDR 6715-15 are 2.145 Mb (43.7% G+C content), with 2,082 predicted genes for strain NIDR 6715-7 and 2,081 predicted genes for strain NIDR 6715-15. The NCTC 10919 genome is 2.145 Mb (43.6% G+C content), with 2,149 predicted genes.

### Data availability.

This whole-genome shotgun project has been deposited in GenBank under the accession numbers CP029490 (SL1), CP029560 (NIDR 6715-7), CP029559 (NIDR 6715-15), and CP029491 (NCTC 10919).
